# Establishment of reference intervals for hematological parameters of adult population in the western region of Saudi Arabia

**DOI:** 10.1371/journal.pone.0281494

**Published:** 2023-02-08

**Authors:** Anwar Borai, Kiyoshi Ichihara, Suhad Bahijri, Abdulaziz Almasoud, Waleed Tamimi, Wail Abdulhadi, Jamil Lingga, Ali Bawazeer, Mohammed Abdelaal, Sultanah Boraie, Abeer Alsofyani, Mohieldin Elsayid, Naif S. Sannan, Ali S. Al-Shareef, Eman Khan, Mohammed Almohammadi

**Affiliations:** 1 King Abdullah International Medical Research Center (KAIMRC), King Abdulaziz Medical City, Ministry of National Guard Health Affairs, King Saud bin Abdulaziz University for Health Sciences (KSAU-HS), Jeddah, Saudi Arabia; 2 Faculty of Health Sciences, Yamaguchi University Graduate School of Medicine, Ube, Japan; 3 Department of Clinical Biochemistry–Faculty of Medicine- King Abdulaziz University, Jeddah, Saudi Arabia; Para Federal University, BRAZIL

## Abstract

**Background:**

Most of hematology laboratories in Saudi Arabia utilize the reference intervals (RIs) provided by instrument manufacturers. This study aimed to define RIs of hematological parameters for adult population in the western region of Saudi Arabia and to explore their specific features from an international perspective.

**Method:**

This study was conducted according to the harmonized protocol of IFCC Committee on RIs and Decision Limits. Blood samples collected from 409 healthy Saudi males and females adults were analyzed for complete blood count (CBC) by using Cell-Dyn Sapphire analyzer and for iron profile by using Architect analyzers. The needs for RIs partitioned by sex and age was based on standard deviation ratio (SDR) and/or bias ratio (BR). RIs were derived parametrically with/without application of the latent abnormal values exclusion method (LAVE).

**Results:**

Based on thresholds of SDR≥0.4 and/or BR≥0.57, RIs were partitioned by sex for red-blood cell count, hemoglobin, hematocrit, red cell distribution width, erythrocyte sedimentation rate, iron, transferrin, ferritin, eosinophil, platelet, plateletcrit, etc. Partitioning by age was not necessary for any of the analytes. LAVE procedure caused appreciable changes in RI limits for most erythrocyte and iron parameters but not for leukocyte parameters. Comparable to other non-IFCC studies on CBC RIs, the RBC and hematocrit (Ht) ranges have shifted to a higher side in both genders. After applying the LAVE method, the male and female RIs for Hb were 4.56 to 6.22 ×10^6^/μL and 3.94 to 5.25 ×10^6^/μL respectively while RIs for Ht were 40.2 to 52.0% and 33.6 to 44.5% respectively.

**Conclusion:**

LAVE method contributed to reducing the influence of latent anemia in deriving RIs for erythrocyte related parameters. Using the up-to-date methods, the RIs of CBC determined specifically for Saudis will help to improve the interpretation of test results in medical decision making.

## Introduction

Clinical interpretations and medical decisions primarily rely on laboratory test results provided by laboratory reports and reference intervals (RIs) [1, 2]. Reference interval defined as a central 95% range of reference values (RVs) from well-defined healthy individuals has been considered to represent the results expected for healthy individuals [3]. It is considered as a golden tool in guiding clinicians to diagnose and monitor diseases precisely, track patients’ responses to treatment, and predict any potential risk factor. RIs often vary with gender, age, geographic area, ethnicity, and other factors leading to variations in RIs among clinical laboratories [2]. Therefore, it is imperative to establish population-specific RIs for major laboratory analytes in each laboratory.

With this background, the Committee on Reference Intervals and Decision Limits (C-RIDL) of the International Federation of Clinical Chemistry and Laboratory Medicine (IFCC) has initiated an international multicenter study aiming to find appropriate RIs for each laboratory by implementing population-specific RIs [4, 5]. Following this initiative, we aimed to establish Saudi-related RIs for basic hematological analytes as a part of a nationwide multicenter study.

A complete blood count (CBC) and other hematology parameters are widely measured in clinical laboratories. Such parameters have clinical values for assessing and diagnosing certain blood conditions and tracking treatment response. Hematological tests and their corresponding RIs are often influenced by age, sex, geographical regions, genetic predisposition, environmental factors, and dietary habits [6, 7]. Additionally, Arab countries, particularly Saudi Arabia, are well known for high prevalence of consanguineous marriages compared with other countries [8–11]. This may have a potential impact on peripheral blood composition [12].

Several studies have been attempted to establish hematological RIs for healthy individuals in many countries, including the Arab gulf [13–17]. There are significant racial and gender differences in almost all studies in calculated RIs for complete blood count parameters demonstrating the necessity to establish population-specific RIs

In Saudi Arabia, a recent multicenter study was conducted in 2019 to derive typical RIs for five hematological analytes. In this study, nearly 1127 healthy volunteers from three Saudi regions were recruited. They found that the calculated RIs for WBC, hemoglobin, platelet, MCV, and neutrophils were vastly lower than those currently used in our clinical laboratories [18]. Three other studies were also carried out in Saudi Arabia, attempting to establish hematological RIs [19–21]. However, these studies were performed without rigorous attempt to exclude individuals with latent diseases, especially iron deficiency states, and without application of up-to-date statistical methods for data analysis and determination of RIs.

Since we have just established the RIs for clinical biochemistry [22] and immunoassay analytes [23] as a part of the global multicenter project coordinated by IFCC/C-RIDL, this study aimed to derive a Saudi-based RIs for hematological parameters again in accordance with the harmonized protocol [4, 5].

## Methods

### Subject recruitment

Following the harmonized IFCC/C-RIDL protocol for recruitment, apparently healthy Saudi citizens were enrolled in this study irrespective of ethnic backgrounds. However, the preference was given to native residents of the Arabian Peninsula. The current study was conducted in the Makkah province located in the western coast of Saudi Arabia. This region includes two large cities, namely Makkah and Jeddah. The study was announced for recruitment by using different tools of communication including direct contact, email distribution, social media, posters or by official letters. The healthy subjects were recruited from different professions and governmental sectors including health, education, military, retirement office, etc.

Ethical approval was obtained from Research Ethics Committee, King Abdullah International Medical Research Center (KAIMRC), King Abdulaziz Medical City, Jeddah, Saudi Arabia (Study number RCJ0212-209).

A total of 409 Saudi adults were recruited aged between 18 and 65 years (51.1% males, 48.9% females). After signing a written informed consent for participation, each volunteer was asked to fill out a questionnaire about lifestyle, ABO blood type, menstrual status, their medical history including recent infection or allergic disorders, family history, whether they took any medication or supplements, and other factors. Height and weight were measured using standardized instruments and techniques, and body mass index (BMI) was computed for each subject. The questionnaire was derived from C-RIDL protocol [4, 5] with some modification to be more suited for Saudi culture. After applying the inclusion and exclusion criteria provided in the C-RIDL protocol, six subjects were excluded because of pathological conditions such as abnormal Hb variants, sickle cell anemia, and leukemia. All abnormalities were confirmed by using more specific tests for the associated conditions such as microscopic examination, sickle cell test, and hemoglobin electrophoresis. Those subjects were informed about their pathological status and referred for treatment. Subjects were not screened for malaria, intestinal parasites, human immunodeficiency virus, and other infectious diseases as their incidences are low in Saudi Arabia,

### Blood collection and handling

The procedure for blood drawing was performed according to the established protocol. The included healthy participants were requested to avoid robust physical activity for three days prior to the blood sampling and to abstain from excessive eating/drinking the night before. Venipuncture was set between 7 and 10 AM following fasting for at least 10 hours. Blood samples were collected in the phlebotomy area of pathology and laboratory medicine at King Abdulaziz Medical City, Jeddah. The participants were living in Jeddah (⁓81%), Makkah (⁓14%), and other cities (⁓5%) of the western region, Saudi Arabia. Jeddah is located on the sea level while Makkah’s altitude is about 200m above the sea level. Participants were asked to remain seated for 30 minutes to avoid any variations in the results owing to postural changes [24]. During the waiting time, blood pressure, height, and weight were taken. Thereafter, 15–20 mL of blood were drawn in six vacutainers. Two EDTA vacutainer tubes were collected for the determination of complete blood count (CBC), glycated hemoglobin (HbA1c), hemoglobin variants and erythrocyte sedimentation rate (ESR). Four plain tubes were collected for chemistry and immunoassay parameters. The measurement of HbA1c was performed for the purpose of future research and to help in detecting abnormal hemoglobin variants. The two EDTA samples were gently inverted 3 to 4 times to make sure EDTA anticoagulant was mixed homogenously with the blood and to prevent blood clotting. After EDTA samples collection they were transported by porters to the main laboratory (hematology section) within 30 minutes for analysis.

For non-CBC measurement, the four collected plain tubes were centrifuged, separated and stored at −80°C until analysis as described in our previously published RI studies for chemistry [22] and immunoassay [23] parameters.

### Measurements

Whole blood samples were examined for CBC using a Cell-Dyn Sapphire Hematology Analyzer (Abbott Diagnostic, USA). Hematological parameters included in the CBC were white blood cell count (WBC), neutrophil absolute count (Neu), lymphocyte absolute count (Lym), monocyte absolute count (Mon), eosinophil absolute count (Eos), basophil absolute count (Bas), neutrophil percentage (Neu%), lymphocyte percentage (Lym%), monocyte percentage (Mon%), basophil percentage (Bas%), eosinophil percentage (Eos%), red blood cell count (RBC), hemoglobin (Hb), hematocrit (Hct), mean corpuscular volume (MCV), mean corpuscular hemoglobin (MCH), mean corpuscular hemoglobin concentration (MCHC), red cell distribution width (RDW), platelet (PLT), mean platelet volume (MPV), platelet distribution width (PDW), and plateletcrit (PCT). TEST 1 (Alifax, Padova, Italy) was used to measure ESR. All measurements were conducted at King Abdulaziz Medical City Laboratory (Jeddah, Saudi Arabia), utilizing the manufacturer’s protocol and reagents, including calibrators and quality controls. For uncovering subjects with latent iron deficiency anemia, iron (Fe), unsaturated iron binding capacity (UIBC), transferrin (TF), and ferritin were measured in the aliquot of sera as described in our first two reports [22] by the auto-analyzer of Architect 16000c for the first three and by 2000i for ferritin.

### Quality control

Quality control (QC) assessments were routinely performed in the laboratories. All laboratory measurements were carried out in accordance with the C-RIDL standardized protocol and standard operation procedure 2 (SOP 2). The department of pathology and laboratory medicine at King Abdulaziz Medical City is accredited by the College of American Pathologists (CAP) laboratory accreditation. The laboratory applies rigorous internal and external quality control regulations. The results of the controls (Bio-Rad Liquichek^TM^) and calibrators were within acceptable limits on every day of the analysis of the research samples.

### Statistical analyses

#### Partitioning criteria

The partitioning method was adapted from Ichihara K, 2008. Briefly, subgrouping of reference values (RVs) by sex and age was based on inter-subgroup variations expressed as a standard deviation (SD) ratio or SDR. The magnitude of between-sex SD (SDsex), between-age SD (SDage), and net-between individual SD (SDindiv) were computed by the two-level nested ANOVA, and SDR for each factor was calculated as a ratio of each SD to the SDindiv: i.e. SDRsex and SDRage. The threshold level of SDR to consider the partitioning of RVs by a given factor was set to 0.40 [25]. In applying the ANOVA, the RVs were divided into four age groups: 18−29, 30−39, 40−49 and 50−65 years. When RVs are highly skewed, like RDW, Eos, Bas, and ESR, their RVs were first logarithmically transformed, and then the SD in the transformed scale was reverse-transformed as described elsewhere [25].

We adopted a secondary criterion termed bias ratio (BR) at LL (or BR_LL_) and at UL (or BR_UL_) to determine the need for partitioning RVs since we occasionally meet situations where the SDR does not represent a true between-subgroup variation at the lower or upper bounds (LL, UL) of the RI [26, 27]. This is defined by the following formula illustrated for sex partitioning:

$$BR_{LL}=\frac{\left|{LL}_{M}-{LL}_{F}\right|}{({UL}_{MF}-{LL}_{MF})/3.92},\;BR_{UL}=\frac{\left|{UL}_{M}-{UL}_{F}\right|}{({UL}_{MF}-{LL}_{MF})/3.92}$$

where subscript M, F, and MF represent male, female, and male + female, respectively.

The denominator reflects the SD comprising the RI, which corresponds to between-individual SD, while the numerator represents the real between-sex bias in LL or UL. BR was also calculated for assessing the effect of the LAVE procedure (see below) as a bias (difference) observed at the LL and UL with/without LAVE.

As a threshold of |BR|, 0.375 was used for assessing the effect of LAVE procedure in analogy to the convention of “allowable analytical bias” at a minimum level. Since the threshold 0.375 was shown to be overly sensitive in determining the need for sex or age partitioning, we used 0.57, which corresponds to the SDR threshold of 0.40. It was based on the relationship of bias and SD for two values x_1_ and x_2_: (SD of x_1_, x_2_ = $\sqrt{2}$ |x_1_−x_2_|) [28]. Hence, the BR threshold of 0.375 was multiplied by $\sqrt{2}$ and set to 0.57 so that it is comparable to the SDR threshold of 0.4: i.e., the denominator of BR and SDR is common or 1/4^th^ of the RI.

#### Derivation of RI

The parametric technique, based on Gaussian transformation of RVs using the two-parameter Box-Cox formula, was utilized to calculate RIs [5]. The bootstrap method was used to calculate the confidence intervals (CIs) of the lower and upper limits (LL and UL): i.e. after the secondary exclusion steps (see below), the final dataset was randomly resampled, allowing replacement until the data size was the same as the source dataset. RIs were then calculated using the resampled dataset. This resampling and recalculation of RIs was repeated 50 times, and CIs for LL and UL were predicted from the repeatedly calculated LLs and ULs of the RIs.

In the process of calculating the RI, we sought to apply LAVE method [29–31] to reduce the influence of latent anemia in determining erythrocyte-related analytes, and to reduce possible influence of latent inflammation in determining leukocyte and platelet-related parameters. The reference tests for use in the procedure were chosen based on Spearman’s correlation coefficient among the parameters (S1 Table). In the initial calculation of the RIs, no exclusion was made, but in the subsequent iterative calculation, subjects with abnormal results among the reference tests (other than the one under calculation) were excluded to obtain refined RIs. The number of iterations was fixed to six times.

## Results

### Recruited subjects

After excluding 6 subjects, a total of 403 recruited subjects participated in the study. The number of male and female participants were 206 and 197, respectively. The mean age ± SD was 39.3±11.6 years for males and 37.4±13.1 years for females with the BMI of 28.7±5.7 kg/m^2^ and 27.8±6.3 kg/m^2^, respectively. The proportions of participants with allergic conditions (rhinitis, atopic dermatitis, or asthma) were 4.4% (males) and 7.1% (females). Current smokers were 58 (29.1%) and 12 (6.4%) of males and females, respectively.

### Source of variation and correlation among analytes

Multiple regression analysis (MRA) was independently performed for each gender by defining RVs of each analyte as an objective variable and a fixed set of explanatory variables (source of variations): age, BMI, smoking (in four levels), and allergy (binary) as shown in Table 1. As a practical level of association for the partial regression coefficients (r_p_), a value of ≥ 0.20 was considered as a significant effect size. An age-related reduction of RVs was observed for RBC, Hb, and Hct solely in males, while the r_p_ value of Ht increased with age in females (r_p_ = 0.208) and decreased in males (r_p_ = −0.200). At the same time, the r_p_ value of ESR was increased with age in males (r_p_ = 0.328) but not in females (r_p_ = −0.008). The r_p_ value of ESR and Neu were increased with BMI in females (r_p_ = 0.371; r_p_ = 0.308) more than males (rp = 0.272; r_p_ = 0.114) respectively (**S1 Fig**). **Table 1** shows that there was no association of smoking (Smk) with WBC in both males (rp = -0.018) and females (rp = 0.053).

**Table 1:** Multiple regression analysis (rp) for different sources of variation on results of CBC and WBC’s in both males and females.

Male	n	R	Age	BMI	Smk	Allergy	Female	n	R	Age	BMI	Smk	Allergy
WBC	196	0.204	-0.148	0.141	-0.018	-0.045	WBC	181	0.256	-0.079	**0.270**	0.053	-0.029
RBC	197	0.325	**-0.275**	0.147	0.094	0.004	RBC	180	0.128	0.128	0.001	0.004	0.003
Hb	197	0.359	**-0.244**	0.029	**0.217**	0.085	Hb	181	0.214	0.178	-0.033	-0.084	0.103
Ht	196	0.292	**-0.200**	0.028	0.178	0.064	Ht	181	0.232	**0.208**	-0.059	-0.092	0.096
MCV	196	0.232	0.170	-0.134	0.088	0.069	MCV	177	0.222	0.108	-0.064	-0.173	0.087
MCH	196	0.185	0.079	-0.096	0.119	0.082	MCH	179	0.172	0.064	-0.003	-0.130	0.088
MCHC	197	0.175	-0.097	-0.001	0.116	0.065	MCHC	181	0.070	-0.017	0.043	-0.029	0.051
RDW	195	0.221	0.053	**0.213**	0.004	0.026	RDW	179	0.325	-0.025	**0.205**	**0.209**	-0.119
PLT	195	0.132	0.061	0.062	-0.061	-0.055	PLT	179	0.154	-0.125	0.157	-0.018	-0.023
MPV	196	0.181	-0.158	-0.002	0.044	0.052	MPV	180	0.116	-0.081	0.041	0.071	0.065
Neu	196	0.180	-0.111	0.114	-0.045	-0.084	Neu	179	0.276	-0.134	**0.308**	-0.029	0.037
Lym	191	0.127	-0.081	0.089	-0.060	0.001	Lym	179	0.174	-0.012	0.129	-0.024	-0.118
Mon	195	0.223	-0.175	0.065	0.108	0.029	Mon	179	0.169	-0.085	0.145	0.075	-0.062
Eos	196	0.240	0.020	0.034	0.137	**0.205**	Eos	180	0.182	-0.025	0.190	0.017	0.025
Bas	196	0.136	-0.090	-0.093	0.016	-0.012	Bas	178	0.187	0.183	-0.117	0.007	-0.092
Neu%	196	0.096	-0.023	0.006	-0.023	-0.092	Neu%	180	0.195	-0.083	**0.201**	0.017	0.082
Lym%	196	0.056	0.013	0.010	-0.040	0.033	Lym%	180	0.175	0.133	-0.162	-0.031	-0.081
Mon%	196	0.190	-0.015	-0.038	0.169	0.066	Mon%	179	0.127	0.017	-0.113	0.068	-0.030
Eos%	196	0.291	0.086	-0.032	0.152	**0.246**	Eos%	180	0.098	0.032	0.065	0.015	0.049
Bas%	196	0.168	-0.044	-0.155	0.021	0.004	Bas%	178	0.235	**0.217**	**-0.214**	-0.006	-0.080
PDW	184	0.085	0.053	0.038	-0.022	-0.037	PDW	155	0.237	0.104	0.012	0.183	-0.081
PCT	185	0.116	-0.095	0.065	-0.010	-0.033	PCT	154	0.232	**-0.227**	**0.222**	-0.030	0.034
ESR	115	0.438	**0.328**	**0.272**	0.003	0.071	ESR	124	0.375	-0.008	**0.371**	0.060	-0.013

BMI, body mass index; Smk, level of smoking-habit; rp, standardized partial regression coefficient. The value of rp of |0.2|or more was considered as significant. Yellow color indicates moderate significant (0.2 ≤ |rp|< 0.30) while green color indicates highly significant (|rp|≥0.3).

Allergy had a slight correlation with Eos level in males (r_p_ = 0.246) more than females (r_p_ = 0.049). S2 Fig shows the magnitude of increased Eos in subjects with allergy compared to those without allergy. In our previous reports about Saudi Ris, we found that by performing the MRA in the same way, the iron markers (Fe, TRF, UIBC, and ferritin) in each gender were not significantly associated with age, BMI, and smoking [22, 23].

**Fig 1:**
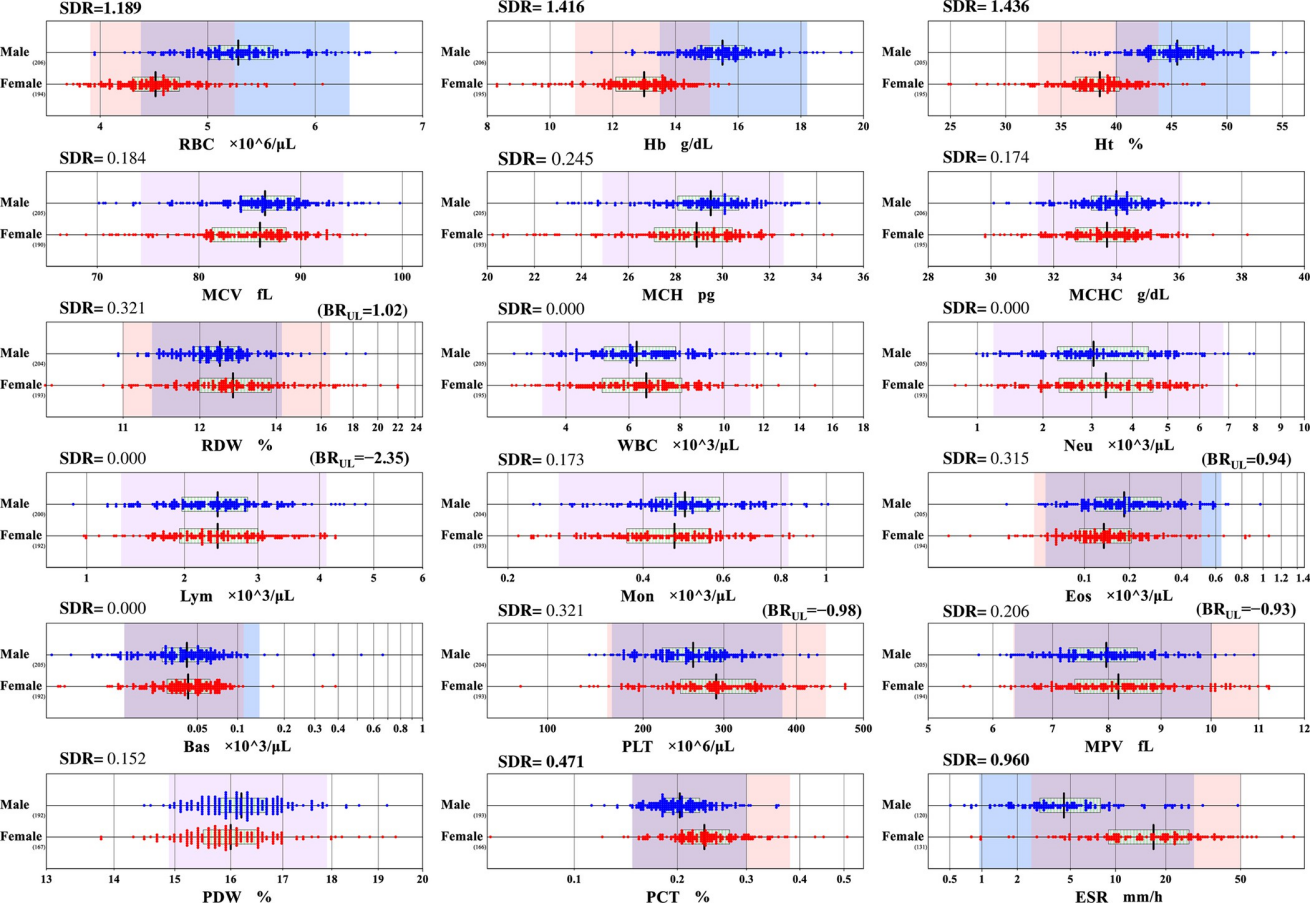
Gender differences in reference values of major hematology parameters. RVs were partitioned by sex (male: M, female: F) for 15 major hematology parameters. Each scattergram’s central vertical bar denotes the median and the box in the middle denotes the mid- fifty percent range of RVs. Subgroups’ data size is presented at the right bottom of the age group labels. The range of the scattergrams might not match the RI since no secondary exclusion was performed from RVs. RIs were determined with the inclusion of a single mean 2.81SD truncation step under the transformed scale.

Using a cut-off of 0.4 as a guide, SDRs have shown that sex was a significant source of variation for RBC, Hb, Hct, PCT and ESR, while PLT and Eos counts had high SDRs but still <0.4 (Table 2). Therefore, partition of RVs by sex was decided for RBC, Hb, Ht, ESR, and PCT (Fig 1). Meanwhile, our previous reports about that the level of SDRsex were significantly high (≥ 0.4) for Fe, TRF, and UIBC [22], and ferritin [23].

**Table 2:** A. Standard deviation ratios (SDR) for (A) the magnitude of sex, age and BMI on hematological analytes and WBC’s in total subjects (B) the magnitude of age on hematological analytes and WBC’s within each of males and females subjects.

**(A)**					**(B)**		
**Analytes**	**N**	**SDRsex**	**SDRage**	**SDRbmi**	**Analytes**	**SDRageM**	**SDRageF**
RBC	391	**1.189**	0.160	0.147	RBC	0.211	0.107
Hb	392	**1.415**	0.198	0.000	Hb	0.256	0.094
Ht	391	**1.436**	0.163	0.000	Ht	0.193	0.117
MCV	386	0.184	0.000	0.218	MCV	0.000	0.109
MCH	389	0.245	0.000	0.184	MCH	0.000	0.000
MCHC	392	0.174	0.100	0.117	MCHC	0.103	0.117
RDW*	388	0.276	0.000	0.202	RDW*	0.000	0.106
WBC*	391	0.000	0.100	0.200	WBC*	0.086	0.167
Neu	389	0.000	0.121	0.157	Neu	0.070	0.187
Lym	383	0.000	0.067	0.114	Lym	0.080	0.108
Mon	388	0.173	0.148	0.142	Mon	0.125	0.183
Eos*	390	**0.315**	0.099	0.000	Eos*	0.138	0.032
Bas*	388	0.000	0.092	0.000	Bas*	0.000	0.202
Neu%	390	0.000	0.129	0.000	Neu%	0.129	0.124
Lym%	390	0.000	0.116	0.000	Lym%	0.132	0.117
Mon%	389	0.260	0.000	0.146	Mon%	0.000	0.000
Eos%*	390	**0.360**	0.033	0.000	Eos%*	0.109	0.000
Bas%*	388	0.000	0.053	0.184	Bas%*	0.000	0.227
PLT	388	**0.321**	0.000	0.000	PLT	0.000	0.000
MPV	390	0.206	0.068	0.000	MPV	0.105	0.037
PDW	350	0.152	0.089	0.000	PDW	0.000	0.168
PCT	350	**0.471**	0.000	0.141	PCT	0.000	0.000
ESR*	249	**0.960**	0.175	**0.302**	ESR*	**0.381**	0.035

SDR, standard deviation ratio; SDRsex, between-sex SDR; SDRage, between-age SDR. Nested ANOVA was applied for between-sex and -age differences. SDR ≥ 0.4 was interpreted as presence of significant between-subgroup differences. The magnitude of SDR more than 0.4 were indicated by 3 colors: 0.0 ≤ SDR<0.4, 0.4≤SDR< 0.6 and 0.6≤SDR.

For all hematology parameters, age and BMI did not appear to be significant sources of variation based on SDR values (Table 2). Hence the age was not considered for partitioning and BMI was not treated as a clue for secondary exclusion of RVs for any analyte. The association study among erythrocyte parameters and iron- markers (iron, Fe; unsaturated iron-binding capacity, UIBC; transferrin, TRF; and ferritin, Ferr) showed generally very high levels of correlations by using the Spearman’s correlation coefficient, especially in females (S1 Table). This result was interpreted as a rationale for applying LAVE method by selecting reference tests from among those analytes. On the other hand, there was no appreciable association of total protein (TP), albumin (Alb), transferrin (TRF), and C-reactive protein (CRP) with leukocytes and platelet related parameters, and thus LAVE method was not applied (S2 Table).

### Derivation of reference intervals

The RIs were derived by using parametric method, with/without application of the LAVE procedure. For selecting reference tests for use in the LAVE procedure, RIs were calculated in two parts, one for erythrocyte-related parameters and the other for leukocyte and platelet related (or non-erythrocyte) parameters as shown in S3 and S4 Tables, respectively. For the former, there were significant associations between iron-markers and erythrocyte parameters. The utility of LAVE was expected for reducing the influence of latent anemia. As reference tests for use in LAVE procedure, seven analytes (Fe, UIBC, transferrin, ferritin, Hb, MCV, and RDW) were empirically chosen to attain an optimal effect for the secondary exclusion. In contrast, no remarkable cross-correlations between inflammatory markers (TP, Alb, transferrin, and CRP) and non-erythrocyte parameters were observed. Hence, the application of LAVE was withheld for them.

We considered the requirement for partitioning RVs by gender based on SDRsex and/or BRs, and the utility of the LAVE method was assessed based on BRs at LL and/or UL. The list of RIs calculated in multiple ways are fully listed in S3 Table for erythrocyte and iron markers and in S4 Table for leukocyte and platelet parameters. It is of note that RIs derived by use of nonparametric method were omitted from the tables.

This was because the RIs by nonparametric method often gave: 1) a wider interval, 2) a broader 90%CI at RI limits, 3) raised UL when the RV distribution showed a prominent tailing to a higher-side (Ferr, Eos%, Bas%, etc), 4) lowered LL when the distribution showed more scatter to a lower-side (Hb, Ht, MCV, MCH, MCHC) as shown in S3 Table. Based on SDRsex and BRs, partitioning by sex was adopted for RBC, Hb, Ht, RDW, and ESR among erythrocyte parameters (S3 Table), and for PLT, MPV, Eos, Eos%, and PCT among non-erythrocyte parameters (S4 Table).

In S3 Table, the comprehensive list of RIs determined by parametric method with/without LAVE are listed for all erythrocyte parameters and iron markers. The effect of the LAVE method was judged from bias ratio at LL or UL (BR_LL_, BR_UL_) using the threshold of 0.375. Hence, the LAVE method was applied in calculating RIs for RBC, Hb, Ht, MCV, MCH, MCHC, RDW, and ESR.

### Sex and LAVE effects on RIs

**Fig 2:**
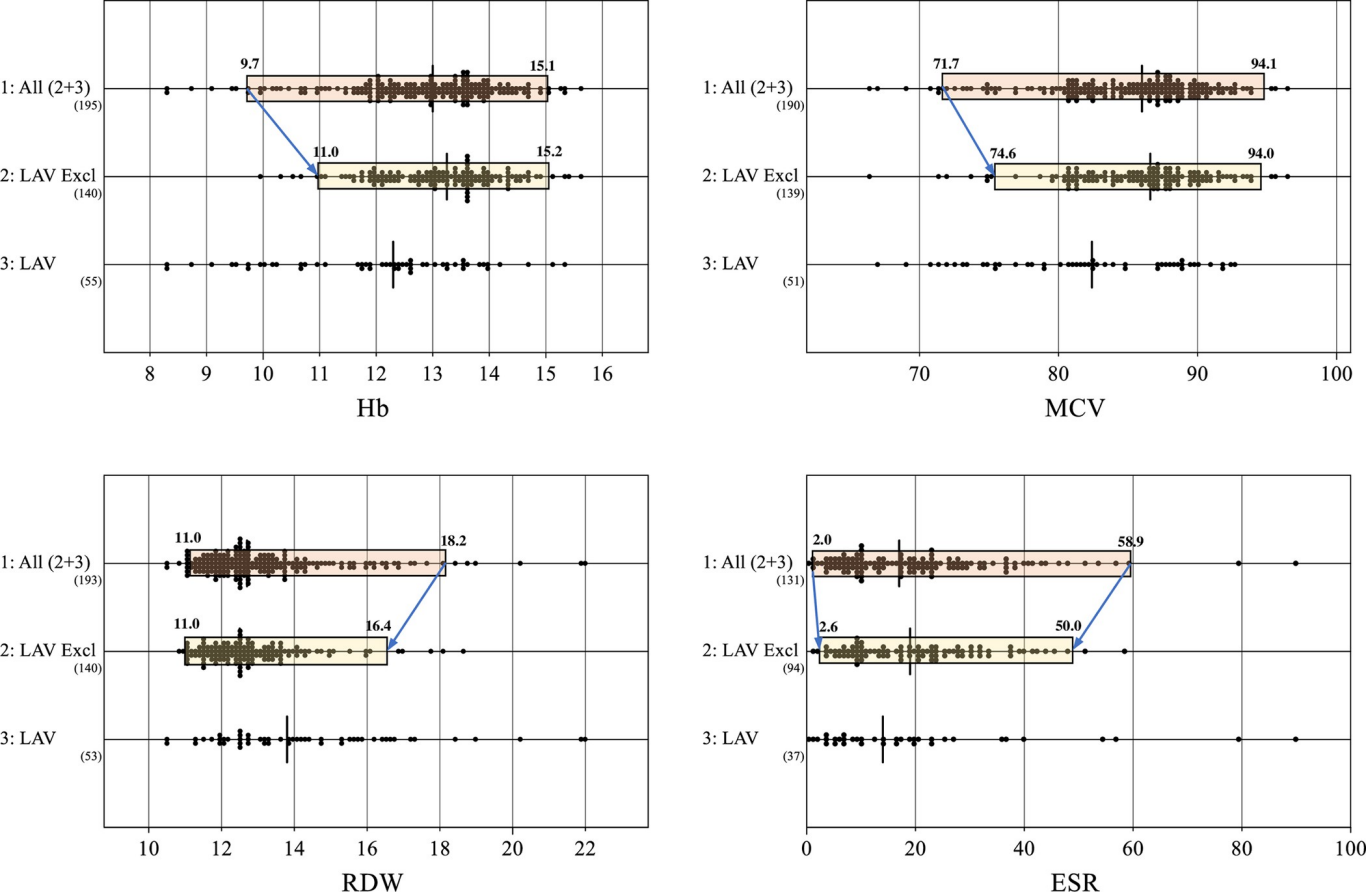
Effect of LAVE method on RIs of anemia-associated parameters. The distribution of female reference values (RVs) for representative anemia-associated markers is shown in three categories: (1) all RVs [1: All (2+3)], (2) RVs that persisted after latent abnormal values exclusion (LAVE) procedure [2: LAV Ex], and (3) the subset of RVs that were excluded by the LAVE procedure [3: LAV]. The RIs determined for the first two categories of RVs are displayed as rectangular areas drawn over the scattergram. The scattergram (1) is divided into scattergrams (2) and (3). In applying LAVE, the subsequent mutually associated reference tests were used for detecting individuals with latent abnormal values: Fe, UIBC, Ferr, Hb, MCV, RDW, and CRP. It is important to note that although there appear to be an excessive number of points outside the RI overlay, values outside the mean 2.81SD were trimmed once during the RI’s derivation.

By applying the LAVE method, the RI of Hb in males was changed from 13.0 to 13.6 g/dL for the LL and from 17.8 to 18.0 g/dL for the UL. In females, the LL was changed from 9.7 to 11.0 g/dL and the UL was changed from 15.1 to 15.2 g/dL. Similarly, appreciable changes in RI limits were also observed for MCV, RDW, and ESR as illustrated in Fig 2.

The RIs for RBC, Hb, Ht, Eos, and Eos% were shifted to a lower side in female than male. The RIs for ESR, PLT, MPV, and PCT were shifted to a higher side in female than male as shown in Fig 1. The final list of RIs selected from S2 and S4 Tables are listed in Table 3.

**Table 3:** The list of RIs summary with 90% CI adopted in consideration of sex with and without LAVE for leukocyte and platelet parameters (3.1), erythrocyte parameters (3.2), and iron markers (3.3).

3.1 Leukcyte and platelet parameters												
					Parametric method without LAVE							
Item	Unit	Sex	LAVE	Age	n	90%CI of LL		LL	Me	UL	90%CI of UL	
WBC	×10^3^/μL	MF	(-)	All	402	3.12	3.63	3.38	6.48	11.31	10.57	12.06
Neu	×10^3^/μL	MF	(-)	All	401	1.10	1.31	1.21	3.15	6.81	6.50	7.12
Lym	×10^3^/μL	MF	(-)	All	394	1.18	1.45	1.31	2.43	4.12	3.93	4.32
Mon	×10^3^/μL	MF	(-)	All	400	0.25	0.27	0.26	0.48	0.83	0.79	0.87
Eos	×10^3^/μL	M	(-)	All	195	0.04	0.07	0.05	0.18	0.64	0.54	0.74
		F	(-)	All	177	0.02	0.06	0.04	0.14	0.51	0.36	0.65
Bas	×10^3^/μL	M	(-)	All	201	0.00	0.01	0.01	0.04	0.14	0.10	0.18
		F	(-)	All	189	0.00	0.01	0.01	0.04	0.11	0.09	0.12
Neu%	%	MF	(-)	All	401	25.3	29.4	27.4	49.9	68.5	67.0	70.0
Lym%	%	MF	(-)	All	399	20.8	25.1	23.0	38.5	58.4	56.5	60.3
Mon%	%	MF	(-)	All	397	3.9	4.7	4.3	7.6	12.0	11.4	12.6
Eos%	%	M	(-)	All	195	0.76	1.03	0.89	2.94	8.54	7.22	9.86
		F	(-)	All	178	0.19	0.84	0.52	2.15	7.21	5.50	8.91
Bas%	%	MF	(-)	All	391	0.07	0.11	0.09	0.62	1.59	1.47	1.72
PLT	×10^6^/μL	M	(-)	All	204	157	173	165	261	380	362	397
		F	(-)	All	193	141	179	160	292	443	423	464
MPV	fL	M	(-)	All	205	6.18	6.53	6.36	7.89	10.01	9.70	10.33
		F	(-)	All	194	6.09	6.58	6.34	8.16	11.00	10.60	11.40
PDW	%	MF	(-)	All	360	14.7	15.0	14.9	16.1	17.9	17.5	18.2
PCT	%	M	(-)	All	192	0.14	0.16	0.15	0.20	0.30	0.28	0.33
		F	(-)	All	163	0.13	0.18	0.15	0.24	0.38	0.30	0.46
**3.2 Erythrocyte parameters**												
					Parametric method with LAVE							
Item	Unit	Sex	LAVE	Age	n	90%CI of LL		LL	Me	UL	90%CI of UL	
RBC	×10^6^/μL	M	(+)	All	137	4.44	4.68	4.56	5.25	6.22	6.09	6.35
		F	(+)	All	147	3.85	4.03	3.94	4.55	5.25	5.13	5.36
Hb	g/dL	M	(+)	All	144	13.2	14.0	13.6	15.6	18.0	17.6	18.5
		F	(+)	All	140	10.6	11.3	11.0	13.1	15.2	14.9	15.5
Ht	%	M	(+)	All	137	39.5	41.0	40.2	45.7	52.0	51.1	52.9
		F	(+)	All	147	32.7	34.5	33.6	38.9	44.5	43.3	45.6
MCV	fL	MF	(+)	All	306	73.4	76.6	75.0	86.5	94.4	93.8	95.1
MCH	Pg	MF	(+)	All	289	24.5	25.5	25.0	29.4	32.5	32.2	32.8
MCHC	g/dL	MF	(+)	All	290	31.2	31.7	31.4	33.9	36.1	35.8	36.4
RDW	%	M	(+)	All	139	11.3	11.4	11.3	12.3	14.1	13.7	14.4
		F	(+)	All	140	10.8	11.3	11.0	12.6	16.4	15.0	17.7
ESR	mm/h	M	(-)	All	120	0.8	1.3	1.0	4.6	29.1	21.8	35.9
		F	(+)	All	94	1.3	4.1	2.6	17.7	50.0	43.0	56.6
**3.3 Iron markers**												
					Parametric method with LAVE							
Item	Unit	Sex	LAVE	Age	n	90%CI of LL		LL	Me	UL	90%CI of UL	
Fe	μmol/L	M	(+)	All	136	8.4	9.5	9.0	15.8	27.4	25.3	29.5
		F	(+)	All	151	3.6	4.9	4.3	11.2	25.6	22.4	28.8
UIBC	μmol/L	M	(+)	All	142	21.8	25.3	23.6	37.5	51.2	49.4	53.0
		F	(+)	All	151	27.5	30.9	29.2	46.2	71.9	68.0	75.8
TRF	g/L	M	(+)	All	141	1.89	2.09	1.99	2.53	3.11	2.99	3.22
		F	(+)	All	151	2.03	2.22	2.13	2.82	3.99	3.81	4.17
Ferr	ng/mL	M	(+)	All	141	3.9	23.0	13.5	110.3	311	252	370
		F	(+)	All	149	3.4	5.5	4.5	23.1	143	98	188

LL, lower limit; UL,upper limit; SDRsex, standard deviation ratio between sex; BR-LL, bias ratio at LL; BR-UL, bias ratio at UL; CI, confidence interval; LAVE, latent abnormal values exclusion method.

### Saudi RIs in comparison to other countries

**Fig 3:**
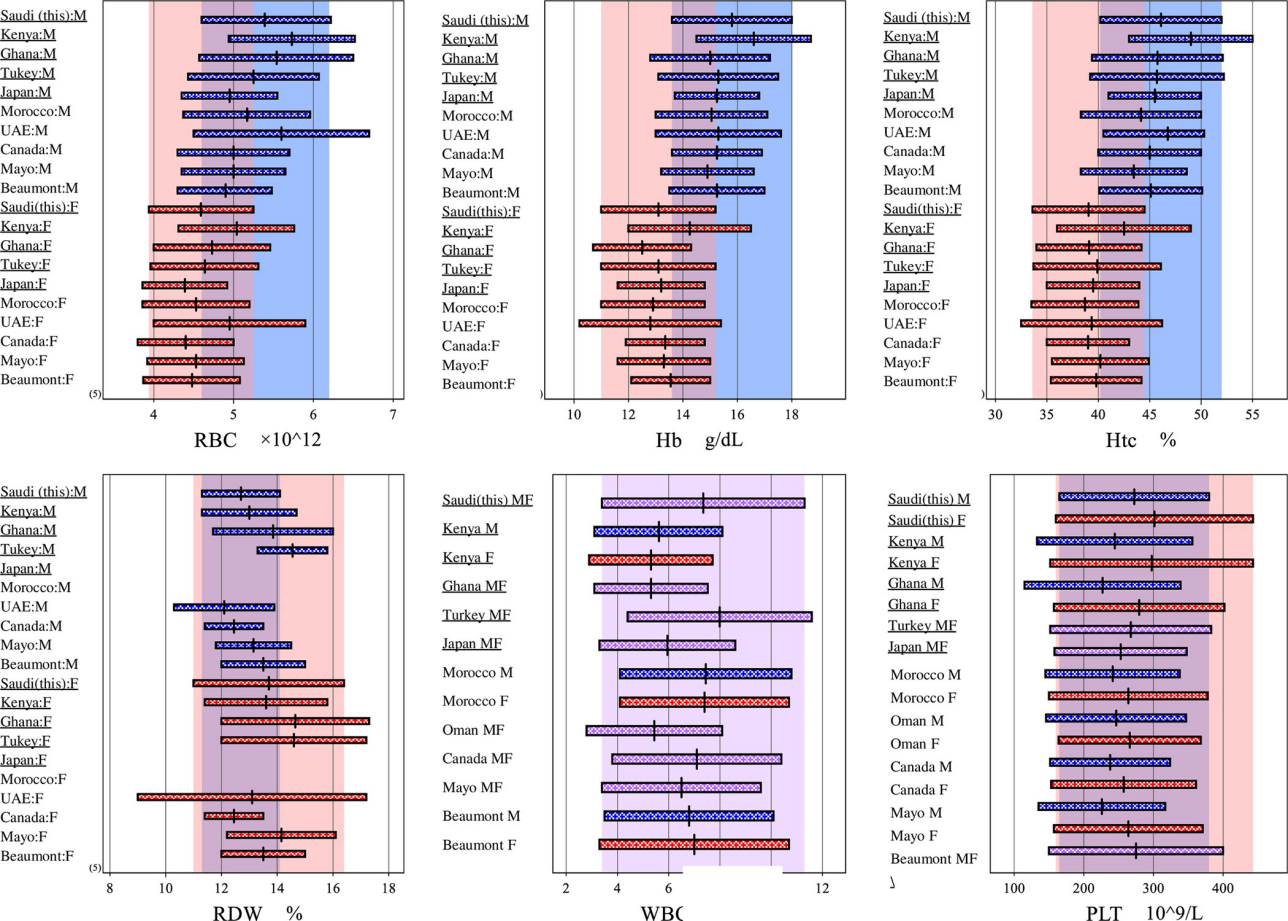
Erythrocyte related RIs of Saudi compared to those reported from other countries. RIs of 10 countries were compared by bar-chart for seven representative analytes that presented features relatively specific to the Saudi population compared to other ethnic groups. The blue, pink, and purple background shades denote the RIs of this study derived for male (M), female (F), and M+F, respectively. Note that the value-assigned serum panel assessed in common was used to standardize all the RIs to make them comparable.

**Fig 3** shows our obtained Saudi RIs in comparison to other countries i.e. Turkey [32], Kenya [33] and Ghana [34] involved in the in the IFCC global project. It seems that countries involved in this multicenter study have the highest upper limits for RBC, Hb and Hct in both males and females. This is because of LAVE effect in excluding subjects with latent anemia in comparison with other countries. Other CBC parameters including RDW, MCV, MCH and MCHC are comparable to all other countries as shown in Fig 3.

## Discussion

For proper derivation of hematology RIs, it is important to analyze factors which can affect test results such as sex, age, ethnicity, BMI, or smoking. They can be used for partitioning RIs, or for secondary exclusion. We used SDR as a primary guide for judging the need of partitioning by sex and age. By setting its threshold at 0.4 as in other studies, SDR for between-age differences (SDRage) was below the threshold in all the hematological parameters we examined, while SDR for between-sex differences (SDRsex) exceeded the level in reference values only for RBC, Hb, Ht, and ESR. In addition to this, the criterion of high |BR| (>0.57), partition of RVs by sex was found to be necessary for RDW, PLT, MPV, Eos, Eos%, and PCT.

Our results are consistent with the previous results in Turkey [32], Ghana [34], Kenya [33], and Japan [35, 36], which used the same statistical scheme. In addition to erythrocyte parameters, between-sex difference was noted for ESR (SDRsex = 0.96). This indicates that it is important to establish separate RIs for males and females and as shown in S1 Fig, the ESR value increased with BMI (SDRmbi = 0.302) in both males and females. This finding is consistent with the finding from a previous study [33]. In addition to this, we found that the SDRsex values were below 0.4 for PLT (0.321) and Eos (0.315), but when between-sex bias ratio at the upper limit (BR_UL_) was calculated, they exceeded the threshold of 0.57. This indicates that it is not appropriate to rely on the SDRsex only, but BR also must be considered to make the final decision for partitioning.

Our results showed that the RIs for CBC and ESR were not appreciably age-related (SDRage<0.4) although partial correlation coefficient by MRA results revealed a weak level of age-related changes for ESR and some of analytes of CBC. Therefore, we did not partition RIs by age in analytes for Saudis.

In this study, we found that Saudi median values for WBC and Neu counts were higher than some African counties e.g. Ghana, Kenya, Uganda, Zimbabwe, and Ethiopia [33, 34, 37–39], but at the same time they were comparable to those in Morocco [40] Turkey [32] and Mayo-clinic laboratory in the United States [41]. The reason for low WBC and Neu counts in Africans compared to Saudis could be due to African-derived null variant of the Duffy antigen receptor for chemokines (DARC-null genotype) as reported in previous studies [33, 34, 42]. Further details about benign ethnic neutropenia in Africans were summarized by Atallah-Yunes et.al, 2019 [42].

The UL of Eos count is less than some African countries such as Kenya, Uganda and Zembabwe but still higher than those in Malaysia [43], Spain [44] and Australia [45]. Eos is known to be elevated with parasitic infection. Therefore, it is probable that the prevalence of parasitic infection and allergic individuals in Saudi population is less common than in African countries, as the total percentage of allergic subjects among Saudi males and females participating in the study were 5.7%. The increased upper limit of Hb in Kenyan males and females (Fig 3) compared to other IFCC countries involved in the global studies can be attributed to the high altitude of Nairobi where the study was conducted. The high altitude can induce erythropoietic process and this consequently will increase the hemoglobin concentration [31, 46]. The extended RIs obtained for the Emiratis were due to the genotypic defined criteria for thalassemia in the recruited subjects [47].

The RI for Fe which was recalculated in this study was consistent with that in our previous report [22, 23], which was calculated from Fe RVs of three cities without reference to CBC test results: i.e., CBC was not tested in two other cities. This comparison of two sets of RIs indicates that RI for Fe did not change much with/without application of the LAVE method considering CBC test results.

The findings from this study agree with a previous study [48] findings as both show that Saudi females have low iron stores and the deficiency of iron is known to be a common cause of thrombocytosis [34, 49]. This kind of association between platelet and Fe levels may explain the high median platelet level in this study for adult Saudi females compared to other countries but at the same time it is compatible to levels for Kenyan females as shown in Fig 3. The scientific mechanism behind this association is not clear, but it is suggested that a decrease in iron can induce thrombocytosis by changing the proliferation and differentiation of pluripotent erythroid and megakaryocyte precursors in bone marrow [49]. Although a recent study showed that thrombocytosis in Saudi females is higher than males (6.34% vs. 1.08%) [50], but still no available study investigated the incidence of thrombocytosis in Saudi females in comparison to other countries. Our study shows that the median value of MPV in Saudi females (8.16 fL) is lower than those in Turkey (8.9fL) and Ghana (10.7 fL). Therefore, we believe that the deficiency of iron in Saudi females and its association with increased platelet count needs further investigation.

In comparison with other RIs studies, we obtained higher UL of Hb (18.0 g/dL) in males and PLT in females (443 ×10^6^/μL) compared to other Arab countries (Oman, Iraq, Qatar, Morocco, Sudan) for Hb and PLT [13, 40, 51–53]. Moreover, S3 Fig shows that our UL of Hb in males and PLT in females are mostly higher than those of four previously published studies on Saudi population (17.9 g/dL, 303×10^9^/μL; [21] 17.2 g/dL, 334×10^9^/μL [54]; 17.5g/dL, 337×10^6^/μL [19]; 18.0g/dL, 367×10^6^/μL [50]) respectively. The reason for the higher-side shift of RVs for hemoglobin and PLT compared in this study could be due to our use of improved statistical approaches including: 1) the parametric method which is robust to extreme values prevalent in skewed distributions unlike nonparametric method [31], and, 2) the LAVE procedure which is effective in reducing the influence of highly prevalent subclinical conditions such as anemia with and without iron deficiency. The effect of parametric and LAVE methods on RBC parameters are shown in S3 Table. By the application of both approaches, the 90% CI of the RI limits became narrower but at the same time are more reliable and efficient specially with the exclusion of recruited subjects with latent diseases [29–31].

In summary, the Saudi healthy subjects recruited in this study were well-defined, as guided in the IFCC/CRIDL harmonized protocol. The application of parametric and LAVE methods helped to improve the reliability and precision of calculated RIs and to reduce the influence of subjects with latent diseases. The limitation of this study was that the healthy subjects were recruited only in the western region of Saudi Arabia. Although some of the recruited subjects were born in other regions including eastern, central, northern, and southern regions, collecting samples from subjects based on other regions would have been more favorable for the study. Nevertheless, it is of note that we experienced no regional difference in RIs of commonly measured chemistry and immunoassay analytes among subjects recruited in three major regions: western, central, and eastern of Saudi Arabia. [22, 23].

## Conclusion

This is the only study in the Arab countries to establish RIs for the hematological parameters using the internationally harmonized protocol with participation of a well-defined healthy Saudi population. Compared to the conventional studies, we believe that the RIs determined in this study are more reliable by using the following up-to-date statistical methods: 1) the parametric method that is less influenced by peripheral extreme values than the nonparametric method, 2) LAVE method that effectively reduced the influence of latent anemia in high prevalence, and 3) combined use of SDR and BR that helped make objective decision for the need of partitioning RVs by sex and age.

## Supporting information

S1 Table Spearman’s correlation coefficients between erythrocyte parameters. Iron related markers (Fe, UIBC, TRF, Ferr) showed variable degrees of closed associations with erthryocyte related parameters. Therefore, reference tests for use in LAVE procedure were set to: Fe, UIBC, TRF, Ferr, Hb, MCV, RDW, and ESR.
(XLSX)

S2 Table Spearman correlation coefficient between leukocyte parameters and inflammatory makers. Inflammatory markers TP, Alb, TRF, and CRP showed no remarkeable association with leukocyte parameters and PLT, whereas there were close associations within-leukocyte parameters. This situation is not appropriate to apply LAVE methods for leukocyte parameters because self-trimming of peripheral values may occur among them.
(XLSX)

S3 Table List of RIs partitioned by sex with/without LAVE method for erhyrocyte-related parameters. LL, lower limit; UL,upper limit; SDRsex, standard deviation ratio between sex; BR-LL, bias ratio at LL; BR-UL, bias ratio at UL; CI, confidence interval; LAVE, latent abnormal values exclusion method. The thresholds for SDR and BR were set to 0.4 and 0.57, respectively. LAVE allowing no abnormal results among Hb, MCV, RDW, Fe, UIBC, TF and Ferr.
(XLSX)

S4 Table List of RIs partitioned by sex for leukocyte and platelet-related parameters. LL, lower limit; UL,upper limit; SDRsex, standard deviation ratio between sex; BR-LL, bias ratio at LL; BR-UL, bias ratio at UL; CI, confidence interval. The thresholds for SDR and BR were set to 0.4 and 0.57, respectively.
(XLSX)

S1 Fig BMI-related change in ESR. (TIF)

S2 Fig Association of allergy with Eos and Eos%. (TIF)

S3 Fig Hematology RIs of Saudi compared to other Saudi studies. Saudi 1, our study; Saudi 2 ref. [51], Saudi 3 ref. [17], Saudi 4 ref. [19], Saudi 5 Ref [52].
(TIF)

S1 Data (XLSX)
