# Supplementary figures and images for: Establishment of reference intervals for hematological parameters of adult population in the western region of Saudi Arabia
Source: PLoS One. 2023 Feb 8;18(2):e0281494. doi: 10.1371/journal.pone.0281494 (PMC9907849; doi:10.1371/journal.pone.0281494)

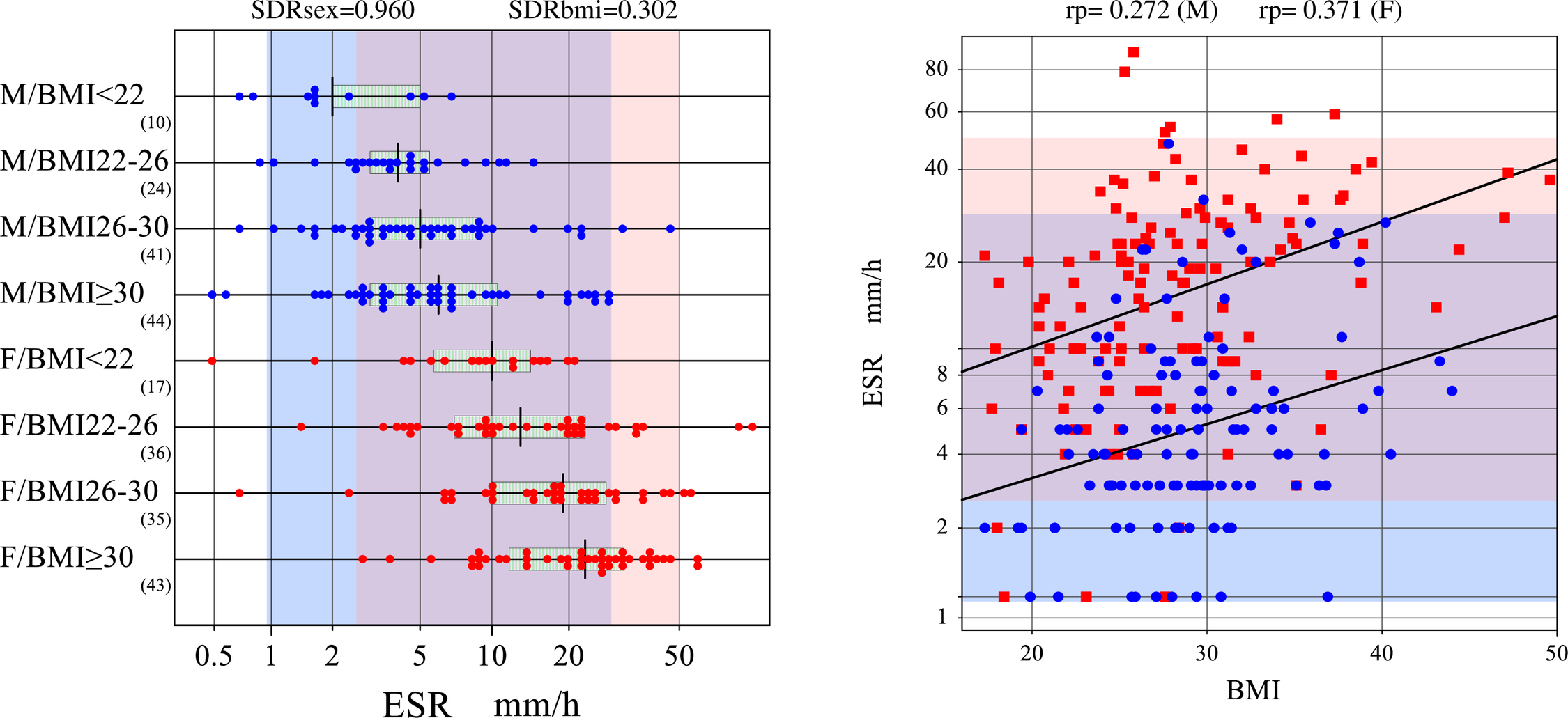

Supplement: S1 Fig — (TIF) [file pone.0281494.s005.tif]

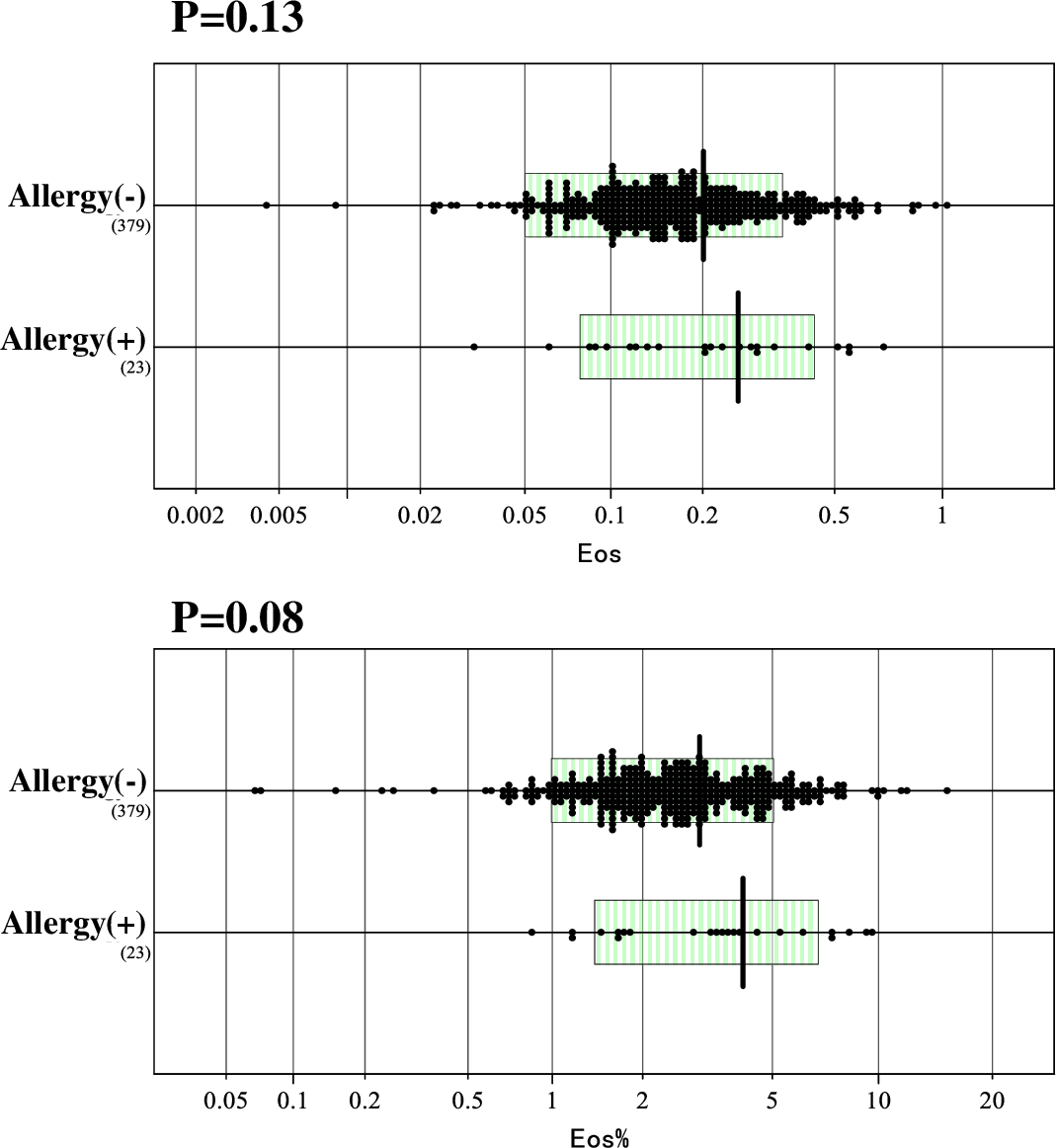

Supplement: S2 Fig — (TIF) [file pone.0281494.s006.tif]

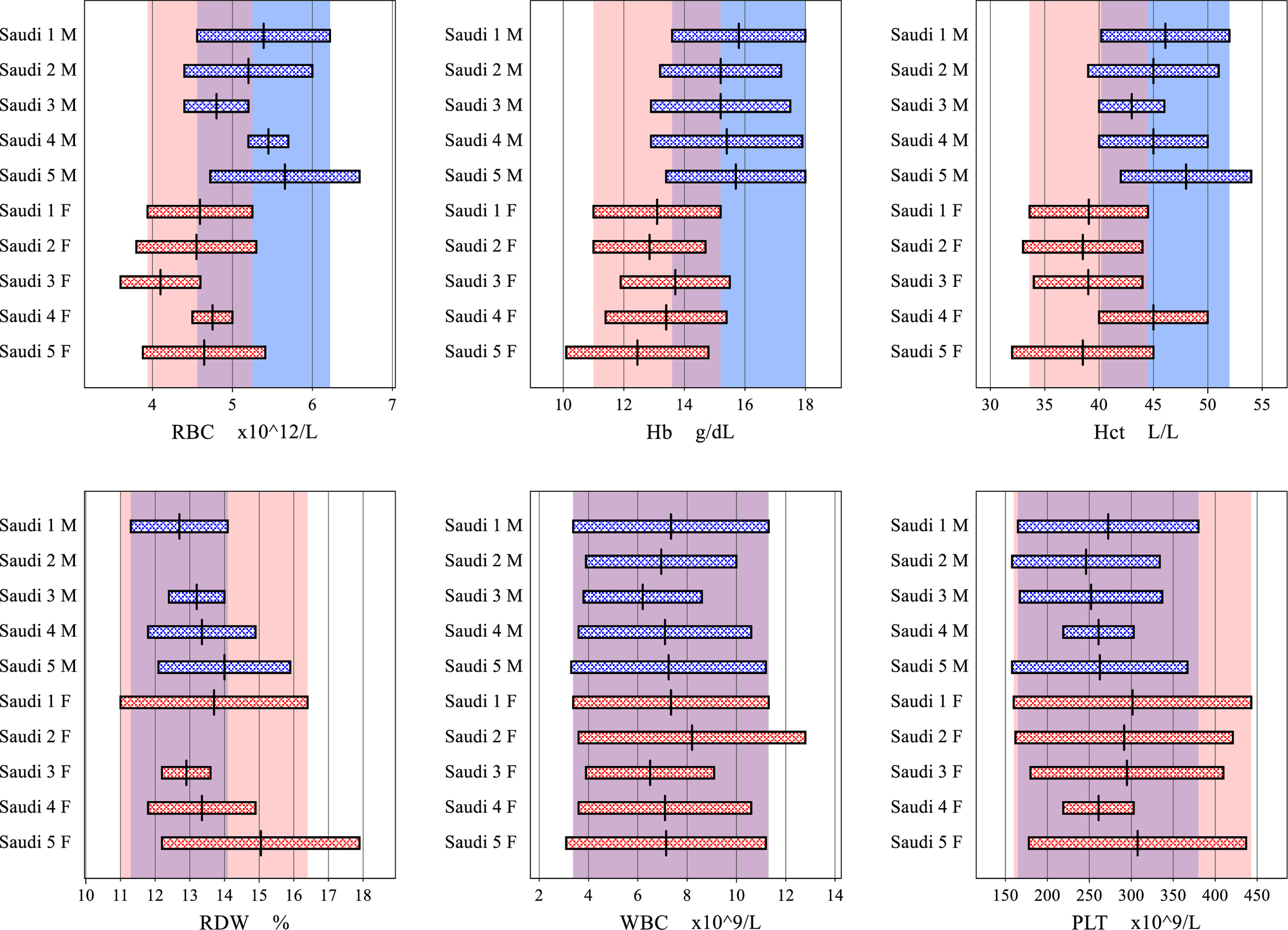

Supplement: S3 Fig — Saudi 1, our study; Saudi 2 ref. [51], Saudi 3 ref. [17], Saudi 4 ref. [19], Saudi 5 Ref [52]. (TIF) [file pone.0281494.s007.tif]
